# Glioblastoma in a female neurofibromatosis 1 patient without IDH1, BRAF V600E, and TERT promoter mutations: A case report

**DOI:** 10.1097/MD.0000000000025346

**Published:** 2021-04-02

**Authors:** Jia-Wei Cai, Xiao-Yong Chen, Jin-Yuan Chen, Zan-Yi Wu, Xi-Yue Wu, Liang-Hong Yu, Hong-Hai You

**Affiliations:** a Department of Neurosurgery; b Department of Ophthalmology, The First Affiliated Hospital of Fujian Medical University, Fuzhou, Fujian, People's Republic of China.

**Keywords:** adult, glioblastoma, neurofibromatosis type 1

## Abstract

**Rationale:**

Glioblastoma is the most lethal and common malignant brain tumor but rare in patients with neurofibromatosis type 1. The clinical findings and pathological findings with gene signatures in female patients have not been well clarified.

**Patient concerns:**

A 51-year-old female patient complained of headache and left limb weakness lasting for 20 days. The patient underwent a cesarean section 20 years ago and hysterectomy 1 year ago because of uterine leiomyomas. Multiple café-au-lait spots and neurofibromas were found over patient's chest, neck, back, and arms. The myodynamia of left distant and proximate epipodite were grade 0 and grade 1 respectively. The myodynamia of lower left limb was grade 3.

**Diagnoses:**

Magnetic resonance imaging revealed a malignant lesion which was most likely a glioblastoma in the right temporo-parietal lobe, approximately 5.6 × 5.9 × 6.9 cm in size with a rounded boundary.

**Interventions:**

A right temporo-parietal craniotomy was performed to resect the space-occupying lesion for gross total removal. Then, the patient received concurrent chemoradiotherapy. Histological examination confirmed a glioblastoma without v-RAF murine sarcoma viral oncogene homolog B1 gene, isocitrate dehydrogenase 1 gene, and telomerase reverse transcriptase gene promoter mutations.

**Outcomes:**

After surgery, the headache was relieved and the muscular strength of left limbs did improve. After receiving the standard treatment regimen, the patient was alive at 13 months follow-up.

**Lessons:**

This is the first reported glioblastoma in female neurofibromatosis type 1 patient without v-RAF murine sarcoma viral oncogene homolog B1 gene, isocitrate dehydrogenase 1 gene, and telomerase reverse transcriptase gene promoter mutations. Tumors in adult patients with these signatures were less aggressive with well-circumscribed border and had long-term survivals which strengthened the evidence that these patients may comprise a unique subset in glioblastoma.

## 1 Introduction

Neurofibromatosis type 1 (NF1) is an autosomal dominant disease which is characteristized by café-au-lait spots and neurofibroma. Global prevalence of NF1 is about 1 in 3500.^[1]^ Low-grade gliomas such as pilocytic astrocytomas and optic glioma are the majority of central nervous system neoplasms found in patients with NF1.^[2]^ Glioblastoma (GBM) is the most lethal and common malignant brain tumor but rare in patients with NF1. The clinical findings and pathological findings with gene signatures in female patients have not been well clarified. We reported a rare case of glioblastoma in a 51-year-old woman with NF1.

## 2 Case information

This case report was approved by the Ethics Committee of the First Affiliated Hospital of Fujian Medical University. Written informed consent was obtained from the patient for publication of this clinical case report.

A 51-year-old female patient complained of headache and left limb weakness lasting for 20 days. The pain was paroxysmal and transient without nausea, vomiting, and dizziness. Painkillers could alleviate the patient's headache but it still reoccurred repeatedly. The limb weakness prevented the female patient from walking and living normally. Otherwise, there was no symptom such as epilepsy or fever occurred in the patient. The patient used to be generally healthy without unhealthy lifestyle. After asking medical history, it was found that the patient underwent a cesarean section 20 years ago and hysterectomy 1 year ago because of uterine leiomyomas. The female patient had no other surgery history and family history of inherited disease.

**Figure 1:**
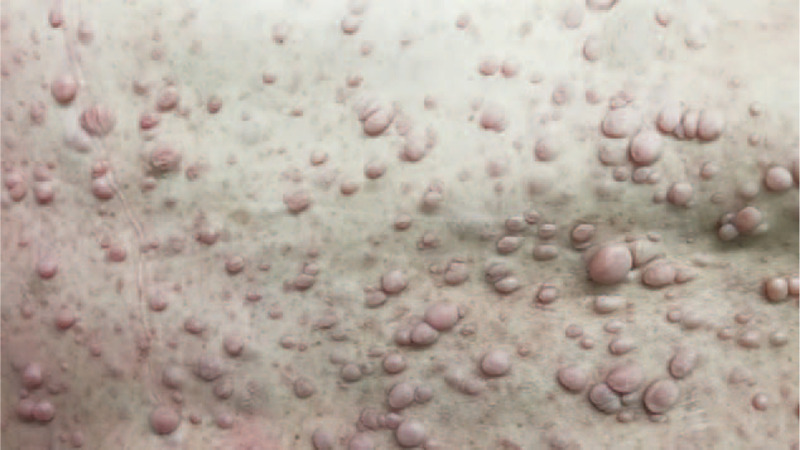
Multiple café-au-lait spots and neurofibromas were found in patient's back.

The admission physical examination showed stable vital signs (body temperature: 36.8°C; heart rate: 86 beats per minute; respiration rate: 19 breaths per minute; blood pressure: 91/50 mm Hg, 1 mm Hg = 1.33 KPa). No obvious abnormality was found in cardiac, lung, and abdominal examination. The patient was in consciousness and cooperative in answering questions. The bilateral pupils were sensitive to light reflection with equal shape and size. The bilateral eyes exhibited free movement. Multiple café-au-lait spots and neurofibromas were found over patient's chest, neck, back, and arms (Fig. 1). The shallow left nasolabial groove, drooping left eyelid and disappearance of forehead line were found in the patient's face. No hypoesthesia or hyperesthesia was found in the patient's face, body, and limbs. The myodynamia of left distant and proximate epipodite were grade 0 and grade 1 respectively. The myodynamia of lower left limb was grade 3. Muscular strength of the other limb and tension of the bilateral limbs were normal. The pathological reflexes were negative.

**Figure 2:**
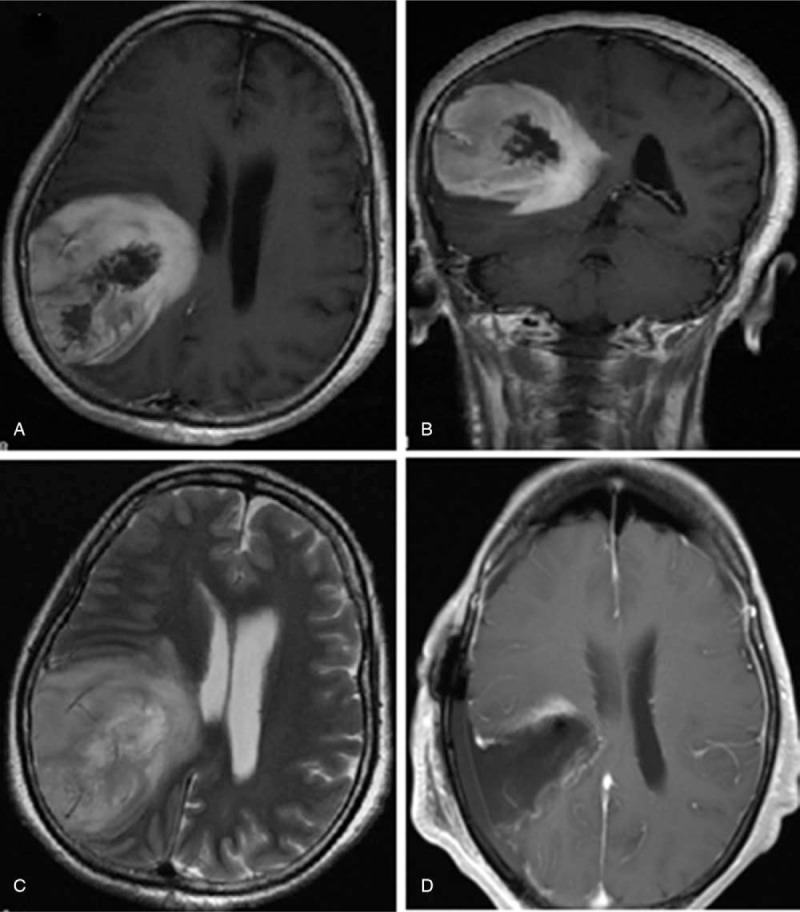
Preoperative and postoperative MRI shows a large lesion and gross total resection in a female adult with neurofibromatosis type 1 and glioblastoma. A. Axial contrast-enhance T1-weighted image shows inhomogeneous enhancement in right temporo-parietal lobe with rounded border. B. Coronal contrast-enhance T1-weighted image shows the tumor with cystic and solid component compress right ventricle. C. T2-weighted image shows a large lesion with peritumoral edema. D. Postoperative MRI demonstrates gross total resection with the tumor.

After admission, laboratory tests including complete blood count, liver function test, renal function test, and other preoperative tests did not show any abnormalities. The electrocardiogram and chest X-ray also revealed normal results. Cranial computed tomography revealed a cystic-solid mass in the right temporo-parietal lobe. Further examination for magnetic resonance imaging (MRI) revealed a malignant lesion which was most likely a glioblastoma in the right temporo-parietal lobe, approximately 5.6 × 5.9 × 6.9 cm in size with a rounded boundary. It has been shown that the tumor with inhomogeneous enhancement compress right ventricle axial and coronal contrast-enhance T1-weighted MRI (Fig. 2. A-B). T2-weighted image shows a large lesion with peritumoral edema (Fig. 2C).

After combining clinical manifestations with radiological examination, the diagnosis was a glioblastoma with neurofibromatosis type 1. Therefore, a right temporo-parietal craniotomy was performed to resect the space-occupying lesion for gross total removal under general anesthesia. The scalp masses were also resected for pathological examination.

**Figure 3:**
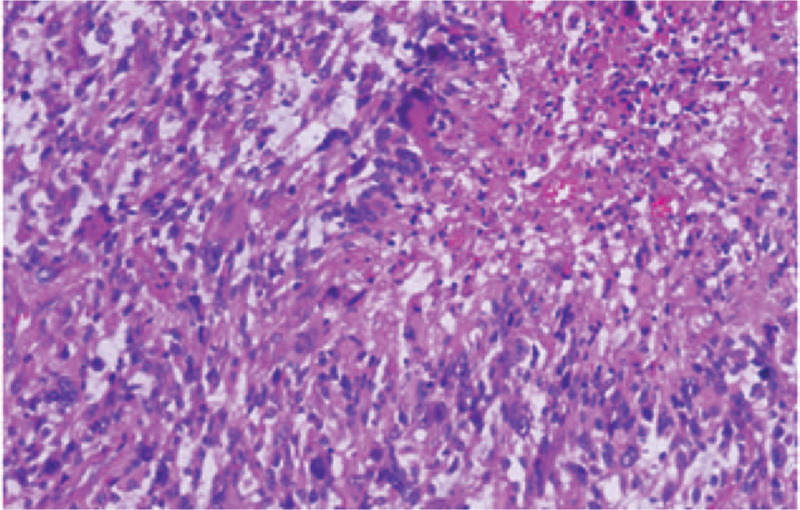
Histological examination shows pathological characteristics of glioblastoma.

Pathological diagnosis of the intracranial lesion showed glioblastoma (Fig. 3). The scalp masses were also confirmed as neurofibroma. Immunohistochemical results of the intracranial specimen showed that glial fibrillary acidic protein, oligodendrocyte lineage transcription factor 2, L1 cell adhesion molecule, S100, and vascular CD34 were positive. The tumor suppressor gene P53 was 80% positive and the methylated level of O6-methylguanine DNA methyltransferase was 4%. The Ki-67 representing cellular marker for proliferation was 30%. Otherwise, there was no mutation in v-RAF murine sarcoma viral oncogene homolog B1 (BRAF) gene, isocitrate dehydrogenase 1 (IDH1) gene, and telomerase reverse transcriptase (TERT) gene promoter.

After surgery, the headache was relieved and the muscular strength of left limbs did improve. The postoperative MRI confirmed gross total resection of the malignant lesion (Fig. 2 D). Then, the patient received concurrent chemoradiotherapy. After receiving the standard treatment regimen, the patient was alive at 13 months follow-up. There were no adverse and unanticipated events.

## 3 Discussion

So far, glioblastoma patients with neurofibromatosis type 1 were rare. Therefore, the distribution of GBM patients with NF1 was unclear which differs from low-grade glioma patients. Infratentorial GBMs represent a small proportion of adult GBMs and the incidence is approximately 1.2%.^[3]^ However, Picart et al reported that NF1 was more frequently occurred in infratentorial GBMs compared with supratentorial GBMs.^[4]^ Based on the current studies, number of NF1 patients with infratentorial GBM was similar from those patients with supratentorial GBMs.^[5,6]^

Table 1 shows the features of previous and our patients of supratentorial glioblastoma with NF1 in adults. Controversies exist in whether NF1 patients with infratentorial GBM require surgery as treatment regimen because of high surgical morbidity.^[4]^ Flower et al reviewed a summary of cerebellar glioblastoma patients with NF1 and reported a case received chemotherapy and radiotherapy only without surgery.^[5]^ The presented case survived 18 months which was ranked third in the group. In addition, the longest survival was achieved by the other patient who received biopsy instead of surgery.^[5,7]^ However, for NF1 patients with supratentorial GBM, surgery with concurrent chemoradiotherapy may still be the best treatment option. Most of them who received the treatment option had a longer survival time than the median time of glioblastoma.^[8]^ The other patients including our case were still alive at the last follow-up.^[9–11]^ In addition, parts of the patients were free of disease for a long time.^[6,10,12]^ However, survival of patients who did not receive standard treatment regimen was short or not available.^[6,13,14]^ Little was known about the relationship between NF1 and GBM. There was a statement that NF1 is a human GBM suppressor gene.^[15]^ In addition, NF1 may be advantageous for survival of glioblastoma in children.^[16]^ While, there was no definitive evidence for adult patients. Comparing with the patients without surgery resection,^[13]^ we thought the standard treatment regimen was still the most effective for adult patients with supratentorial glioblastoma and NF1.

**Table 1:** Summary of adult NF1 patients with supratentorial glioblastoma.

No.	Author	Age/gender	Location	Treatment	Survival
1	Miaux et al (1997)^[18]^	32/F	Occipital	NA	NA
2	Pal et al (2001)^[19]^	37/F	Occipital	No treatment	Finding at autopsy
3	Miyata et al (2005)^[10]^	30/F	Frontal	SR+RT+CT	10 months +
4	Hakan and Aker (2008)^[17]^	28/F	Frontal	SR+RT+CT	41 months
5	Mehta et al (2008)^[13]^	63/M	Parietal	Bp	2 months
6	Jeong and Yee (2014)^[9]^	32/M	Frontal	SR+RT+CT	9 months +
7	Varghese and Abdul Jalal (2015)^[20]^	60/M	Frontal	SR+RT+CT	NA
8	Shibahara et al (2018)^[12]^	52/M	Occipital	SR+RT+CT	49 months
9	Shibahara et al (2018)^[12]^	34/M	Frontal	SR+RT+CT	106 months +
10	Shibahara et al (2018)^[12]^	28/M	Insula	SR+RT+CT	60 months +
11	Shibahara et al (2018)^[12]^	53/M	Frontal	SR+RT+CT	87 months +
12	Singla et al (2018)^[11]^	25/M	Frontal	SR+RT+CT	12 months +
13	Narasimhaiah et al (2019)^[6]^	21/F	Frontal	SR+RT+CT	37 months +
14	Narasimhaiah et al (2019)^[6]^	26/M	Corpus callosum	SR	ND
15	Wing et al (2019)^[14]^	27/M	Multiple	Bp+RT+CT	ND
16	Present	51/F	Temporo-parietal	SR+RT+CT	9 month +

F = female, M = male, NA = not available, SR = surgical resection, RT = radiotherapy, CT = chemotherapy.

Globally, glioblastoma with neurofibromatosis type 1 were a rare disease as the publicly reported cases was approximately 30.^[5,6,11,14,17–20]^ After excluding children patients and infratentorial GBM patients, there was about only half cases left. The reported cases presented different characteristics with incomplete data. Wong et al claimed that KMT2B mutations may be somatic oncogenic events in patients with NF1 and glioblastoma.^[14]^ Singla et al reported a patient with NF1 and Pleomorphic Xanthoastrocytoma converted to GBM after 2 years follow-up.^[11]^ Ameratunga et al presented a case with NF1 and GBM benefiting from MEK inhibitor.^[21]^ Bevacizumab has been proved effective in adult NF1 patients with recurrent high-grade gliomas.^[22]^ However, Ullrich et al reported moyamoya syndrome occurring on a child with NF1 and GBM after receiving angiogenesis inhibitor treatment.^[23]^ Fully understanding clinical and pathological characteristics may help us to explore the relationship between NF1 and GBM and improve prognosis of those patients. Table 2 shows the pathological information in 9 adult cases with supratentorial glioblastoma and NF1. Most of the cases did not reveal pathological characteristics in detail. Shibahara et al reported GBM in 4 NF1 patients without IDH1, BRAF V600E, and TERT promoter mutations.^[12]^ All of them harboured intracranial well-circumscribed tumor and received surgical resection and concurrent chemoradiotherapy. Three of them were still alive at 5 years follow-up. The author proposed that these tumors containing unique pathological features should be distinguished from typical GBM of IDH wildtype. Our case showed similar characteristics above. To our best knowledge, it was the first case report of GBM in female NF1 patient without IDH1, BRAF V600E, and TERT promoter mutations. Also, the tumor was less aggressive with well-circumscribed border. After receiving the standard treatment regimen, the patient was alive at 13 months follow-up. It strengthened the evidence that these patients may comprise a unique subset in GBM and increased the possibility for further study. However, our findings were limited with incomplete sample.

**Table 2:** Pathological features of adult NF1 patients with supratentorial glioblastoma.

No.	Author	GFAP	S100	EGFR amplification, MLPA	Ki-67	P53	MGMT	IDH1 mutation	BRAF V600E mutation	TERT promoter mutation	ATRX mutation
1	Miyata et al (2005)^[10]^	+	+	NA	58%	NA	NA	NA	NA	NA	NA
2	Jeong and Yee (2014)^[9]^	+	NA	–	10%	NA	–	NA	NA	NA	NA
3	Shibahara et al (2018)^[12]^	NA	NA	–	20%	<10%	Low	–	–	–	Retain
4	Shibahara et al (2018)^[12]^	NA	NA	–	38%	<20%	Low	–	–	–	Retain
5	Shibahara et al (2018)^[12]^	NA	NA	–	40%	<10%	High	–	–	–	Retain
6	Shibahara et al (2018)^[12]^	NA	NA	–	20%	<10%	High	–	–	–	Retain
7	Narasimhaiah et al (2019)^[6]^	+	+	NA	25–30%	+	NA	–	NA	NA	–
8	Narasimhaiah et al (2019)^[6]^	+	NA	NA	25–30%	+	NA	–	NA	NA	+
9	Wing et al (2019)^[14]^	NA	NA	NA	NA	NA	NA	+	NA	NA	NA
10	Present	+	+	NA	30%	80%	Low	–	–	–	NA

+ = positive or mutated, - = negative or without, NA = not available, GFAP = glial fibrillary acidic protein, EGFR = epidermal growth factor receptor, MLPA = Multiple ligation-dependent probe amplification, MGMT = O6-methylguanine DNA methyltransferase, IDH1 = isocitrate dehydrogenase 1, BRAF = v-RAF murine sarcoma viral oncogene homolog B1, TERT = telomerase reverse transcriptase. ATRX = alpha-thalassemia/mental retardation syndrome X-linked.

## 4 Conclusion

This is the first reported GBM in female NF1 patient without IDH1, BRAF V600E, and TERT promoter mutations. Further large-scale study is needed to determine whether these patients were a distinctive subset in GBM of IDH wildtype and their relationship with clinical outcome.

## Author contributions

**Conceptualization:** Xi-Yue Wu, Liang-Hong Yu.

**Data curation:** Zan-Yi Wu, Liang-Hong Yu.

**Formal analysis:** Zan-Yi Wu, Xi-Yue Wu, Liang-Hong Yu.

**Funding acquisition:** Jia-Wei Cai, Liang-Hong Yu.

**Investigation:** Liang-Hong Yu.

**Methodology:** Liang-Hong Yu.

**Project administration:** Liang-Hong Yu, Hong-Hai You.

**Resources:** Hong-Hai You.

**Software:** Hong-Hai You.

**Supervision:** Hong-Hai You.

**Validation:** Hong-Hai You.

**Visualization:** Hong-Hai You.

**Writing – original draft:** Jia-Wei Cai, Xiao-Yong Chen, Jin-Yuan Chen, Hong-Hai You.

**Writing – review & editing:** Jin-Yuan Chen, Liang-Hong Yu, Hong-Hai You.
